# Impact of quantitative dietary guidance on postoperative outcomes in patients undergoing transjugular intrahepatic portosystemic shunt surgery: a retrospective cohort study

**DOI:** 10.3389/fnut.2026.1671392

**Published:** 2026-02-11

**Authors:** Yue Xu, Qin Yin, Jiangqiang Xiao, Qing Zhao, Xiaotian Chen, Ming Zhang, Bo Gao

**Affiliations:** 1 Department of Clinical Nutrition, Nanjing Drum Tower Hospital, Affiliated Hospital of Medical School, Nanjing University, Nanjing, China; 2 Department of Gastroenterology, Nanjing Drum Tower Hospital, Affiliated Hospital of Medical School, Nanjing University, Nanjing, China; 3 Department of Gastroenterology, Beijing Ditan Hospital, Capital Medical University, Beijing, China

**Keywords:** TIPS, quantitative dietary guidance, cirrhosis, hepatic encephalopathy, mortality

## Abstract

**Background:**

The optimal nutritional management strategy after transjugular intrahepatic portosystemic shunt (TIPS) procedure in cirrhotic patients remained controversial. A quantitative dietary intervention approach was developed for patients in the post-TIPS period, and its impact on clinical outcomes was evaluated in this study.

**Methods:**

This study was a retrospective, non-randomized controlled cohort study. A total of 92 cirrhotic patients who underwent TIPS were enrolled. Following TIPS, patients were categorized into two groups according to whether they received TIPS-oriented quantitative dietary intervention during hospitalization. The quantitative dietary guidance group received individualized and quantitative dietary instructions after TIPS, and the usual care group served as control. The primary endpoint was death, and the secondary endpoint was overt hepatic encephalopathy (OHE) occurrence. Kaplan–Meier survival analysis and Cox proportional hazards regression models were used to evaluate the association between quantitative dietary intervention and outcomes.

**Results:**

The quantitative dietary guidance group (*n*=54) showed significantly lower mortality rates (5.56% vs. 21.05%, *p*=0.05) and OHE occurrence (12.96% vs. 36.84%, *p*=0.01) during follow-up than the usual care group (*n*=38). Liver-related mortality was also significantly lower in the quantitative dietary guidance group (1.85% vs. 15.79%, *p*=0.04). Multivariate Cox regression analysis demonstrated that the dietary intervention was independently associated with lower liver-related mortality risk (HR 0.09, 95% CI 0.01–0.75, *p*=0.03) and OHE risk (HR 0.34, 95% CI 0.14–0.85, *p*=0.02). Survival analysis demonstrated that the OHE probability was significantly lower in the quantitative dietary guidance group compared to the usual care group (HR 0.32, 95% CI 0.13–0.77, *p*=0.01), as was liver-related survival (HR 0.13, 95% CI 0.02–0.66, *p*=0.03).

**Conclusion:**

A structured quantitative dietary intake protocol following the TIPS procedure could improve survival rates and reduce the incidence of HE. These findings highlighted the importance of TIPS-oriented nutritional management for cirrhotic patients.

## 1 Introduction

Liver cirrhosis was a chronic liver disease that could progress to the decompensated stage, leading to life-threatening complications, including esophagogastric variceal bleeding secondary to portal hypertension (1, 2). The transjugular intrahepatic portosystemic shunt (TIPS) procedure, which created a side-to-side shunt between the portal and hepatic veins, had been shown to reduce portal pressure effectively and was widely utilized in clinical practice for the management of portal hypertension and its complications (1). Despite its efficacy, the management of TIPS-associated complications remained difficult in routine clinical practice. The metabolic disturbances caused by hemodynamic changes following TIPS placement represent a significant clinical challenge.

Hepatic encephalopathy (HE) emerged as one of the most prevalent complications following the TIPS procedure, with reported incidence ranging from 35 to 50% (3, 4). While the exact pathogenesis of HE remained incompletely understood, hyperammonemia was consistently identified as a crucial pathophysiological factor (5). Ammonia, predominantly produced in the gastrointestinal tract as a byproduct of protein metabolism, accumulated systemically when the detoxification capacity of the liver was impaired, resulting in neurotoxic effects on the central nervous system (6, 7). Patients exhibited transient hepatic dysfunction post-TIPS, which further compromised ammonia clearance. Additionally, the created portosystemic shunt permitted gut-derived nitrogenous substances to bypass hepatic metabolism and enter systemic circulation directly, thereby elevating the risk of HE (8, 9). Although TIPS alleviated symptoms and could improve dietary intake, malnutrition remained common postoperatively due to suboptimal dietary patterns and concerns about HE. Systematic data on malnutrition incidence after TIPS were limited, but it was expected to be similar to that in patients with chronic liver disease (20–50%) (10). Malnutrition and sarcopenia in this population were closely associated with HE and overall prognosis.

Accordingly, comprehensive optimal perioperative management to reduce the incidence of HE is of critical importance in patients undergoing TIPS. Protein restriction had been traditionally implemented for HE prophylaxis in clinical practice (11). However, evidence suggested that excessive protein and caloric restriction potentially worsened outcomes in hospitalized HE patients (12). The study by Abou-Assi et al. (13) demonstrated that while protein restriction effectively reduced ammonia generation, overly aggressive limitation exacerbated hepatic insufficiency and precipitated additional complications. Moderate protein intake proved essential for preserving hepatic function and preventing HE. A randomized controlled trial indicated that standard protein diets might offer superior outcomes compared to protein-restricted regimens in HE management through maintaining nutritional status, preventing muscle wasting, and enhancing ammonia metabolism (14). Therefore, appropriate nutritional and dietary guidance is essential.

Current dietary guidelines provided guidance only for chronic liver disease and severe HE status, and there was no research on protein rationing after the TIPS procedure. The uniqueness and innovation of our study lay in the quantitative dietary design and evaluation of its impact on clinical outcomes in post-TIPS patients, aiming to provide evidence-based guidance for clinical practice.

## 2 Methods

### 2.1 Participants

This study enrolled patients with liver cirrhosis who underwent TIPS in Nanjing Drum Tower Hospital during April 2022 and September 2023. A total of 92 cirrhotic patients who underwent TIPS were included in the final study. Data were retrospectively analyzed from our prospectively performed nutritional management routine in our hospital.

Inclusion criteria were as follows: age between 18 and 85years; a definitive diagnosis of liver cirrhosis; successfully underwent TIPS at our hospital; voluntarily participated in this study. Exclusion criteria were as follows: severe HE, severe cardiac dysfunction, or renal insufficiency (eGFR <30mL/min/1.73m^2^); obesity defined as BMI >30 kg/m^2^, excluding the confounding effects of ascites; underwent TIPS recanalization; poor compliance; lost to follow up.

This study was approved by the Clinical Research Ethics Committee of Nanjing Drum Tower Hospital (2022-599-03). Written informed consent was obtained from all participants.

### 2.2 Baseline characteristics

Demographic characteristics (age, height, weight, etc.) were obtained from medical records. Fasting blood samples were collected during initial hospitalization to assess potential covariates influencing study outcomes. In the case of fluid retention, the actual body weight (BW) needed to be adjusted to estimate the dry body weight. Dry body weight was calculated based on the BW and a standardized deduction according to the severity of ascites (mild: 5%; moderate: 10%; severe: 15%), with an additional 5% deduction for the presence of bilateral limb edema. The adjusted BMI was calculated as dividing the dry body weight by the square of the body height (kg/m^2^) (15, 16). Child–Pugh score and model for end-stage liver disease (MELD) were adopted in the present study to assess liver function and prognosis (17). Disease-related information and TIPS procedure information were also documented.

### 2.3 CT anthropometric measurements

Participants enrolled underwent abdominal CT scans during admission. Skeletal muscle mass measurement was performed following protocols based on the previous study (18). A single axial image at the level of the third lumbar vertebra (L3) was selected for analysis. The skeletal muscle area was manually delineated and segmented using MATLAB software (MathWorks, United States), applying Hounsfield unit (HU) thresholds of −29 to +150 to identify skeletal muscle tissue. The major muscle groups included in the L3 region were the psoas, erector spinae, quadratus lumborum, transversus abdominis, external oblique, internal oblique, and rectus abdominis. The cross-sectional area of the skeletal muscle (cm^2^) was calculated by summing the relevant pixels. The skeletal muscle mass index (SMI) was calculated by normalizing the muscle area to squared height (cm^2^/m^2^) and was used to assess sarcopenia (19, 20). Based on previous studies on Asian patients with cirrhosis, sarcopenia was defined in the present study as SMI ≤42cm^2^/m^2^ for males and ≤38cm^2^/m^2^ for females (21, 22).

### 2.4 TIPS procedure

As with previous studies, RUPS-100 (COOK; Indiana, United States) was used in a transjugular venous approach to catheterize the right or middle hepatic vein under local anesthesia (23, 24). Indirect portography was conducted through the superior mesenteric or splenic artery to delineate portal venous anatomy. An intrahepatic tract was established by puncturing from the hepatic vein to a portal vein branch. After successful access, a polytetrafluoroethylene-covered stent of 6mm or 8mm was deployed to create the shunt. Balloon dilation was applied to achieve the target stent diameter. Portal pressure gradient (PPG) was measured before and after stent placement, with hemodynamic success criteria defined as a decrease in PPG to <12mmHg or ≥50% from baseline. The TIPS procedure was performed by a team of experienced gastroenterologists. Continuous non-invasive hemodynamic monitoring, including blood pressure and heart rate, was maintained throughout the surgery to assess circulatory stability.

### 2.5 Dietary intervention for the post-TIPS period

Individualized quantitative dietary guidance was administered to patients according to their personal preferences and in alignment with the routine ward visit schedule of the clinical dietitians. A dedicated clinical nutritionist who was in charge of liver nutrition support conducted scheduled weekly ward rounds and initiated quantitative post-TIPS dietary counselling after admission before surgery. Patients who underwent TIPS were categorized into two groups based on whether an individualized quantitative dietary intervention was provided. The nutritional intervention adhered to the hospital’s standardized protocol for clinical nutrition management.

The quantitative dietary plans were developed based on ESPEN guidelines for energy and slightly modified for protein intake in patients with chronic liver disease after TIPS (25). We adopted a protein target of 1.0g/kg/day in the early post-TIPS period because portal-systemic shunting transiently impaired ammonia detoxification, and slightly excessive protein intake may precipitate HE. Our previous preliminary unpublished evidence based on clinical observation also showed that patients who developed overt HE (OHE) had higher protein intake than those without HE during the first month after TIPS, supporting the rationale for a conservative short-term target. Therefore, we developed a novel, quantitative dietary protocol specifically for the short-term post-TIPS period. After approximately 1month, once physiological compensation was restored, the protein intake was increased to the guideline-recommended level of 1.2–1.5g/kg/day.

In details, patients in the quantitative dietary guidance group received a tailored diet plan for the first month after the TIPS procedure, with an energy intake of approximately 35kcal/kg/day and protein intake of 1.0g/kg/day. Details of the dietary plan within the first month after TIPS were available in the Supplementary Table 1. As part of the standardized protocol, oral nutritional supplement (ONS) was administered to ensure adequate energy and high-quality protein intake among patients in the quantitative dietary guidance group. The ONS regimen consisted of 500mL per day, administered in three divided servings between meals. This daily dosage provided 16g of soy protein and 375kcal, with the total energy comprised of carbohydrates (45%), fat (38%), and protein (17%). Soy protein served as the exclusive source of protein in the ONS regimen, which was naturally rich in branched-chain amino acids. After the first postoperative month, as patients gradually achieved metabolic compensation and their condition stabilized, their dietary intake was adjusted to the standard regimen for chronic liver disease as the ESPEN guideline recommended (approximately 35kcal/kg/day for energy intake, 1.2–1.5g/kg/day for protein intake) (25). In cases of severe HE, patients were advised to discontinue the dietary protocol and seek immediate medical care. The dietary intervention may be resumed once symptoms had resolved and clinical reassessment confirmed stability. The clinical dietitian in charge formulated individualized quantitative meal plans based on patient-specific parameters (e.g., body height and weight, relevant clinical data) and provided the plans to participants in printed form. Patients were instructed to use a kitchen digital scale to measure and adhere to the prescribed daily food quantities. Patients were committed to compliance after discharge from the hospital and followed the plan.

Patients in the usual care group underwent routine nutritional risk screening using the Nutritional Risk Screening 2002 (NRS 2002) tool at admission and received general dietary advice from ward nurses, as was standard for all in-hospital patients. However, they did not receive a quantitative dietary plan designed for the post-TIPS period from a dietitian. Specifically, no individualized food portion lists or quantitative meal plans were provided.

### 2.6 Follow-up

Post-discharge clinical outcomes were collected via telephone interviews, outpatient follow-ups, or hospital admission records. The primary endpoint was the event of death from all causes of illness, or the end date of February, 2025. The secondary endpoint was the onset of OHE. OHE manifested as personality alterations, disorientation, acute confusional states, and could progress to stupor or coma, corresponding to grades II–IV of the West Haven criteria (26, 27). Survival state and post-procedure adverse events were documented. Compliance with the household dietary regimen was also evaluated.

### 2.7 Statistical analysis

All statistical analyses were performed using SPSS software (version 26.0; IBM, United States), and graphical presentations were generated with Prism version 8.0.2 (GraphPad). Continuous variables were expressed as mean±standard deviation or median (interquartile range, 25th–75th percentiles) as appropriate. Between-group comparisons were performed using the independent Student’s *t*-test or Mann–Whitney *U* test for continuous variables, while categorical variables were analyzed using the *χ*^2^ test.

Univariate and multivariate Cox proportional hazards regression models were conducted to estimate hazard ratios (HRs) with corresponding 95% confidence intervals (CIs) to assess the associations of quantitative dietary intervention with clinical outcomes. The comparison of clinical outcomes between the two groups was illustrated by Kaplan–Meier survival analysis with the log-rank test.

For the multivariate Cox regression model, we employed a backward stepwise selection approach with a removal criterion of *p*>0.10 based on likelihood ratio tests. All statistical tests were two-tailed, and a *p*<0.05 was considered statistically significant.

## 3 Results

### 3.1 Baseline characteristics of the study participants

**Figure 1:**
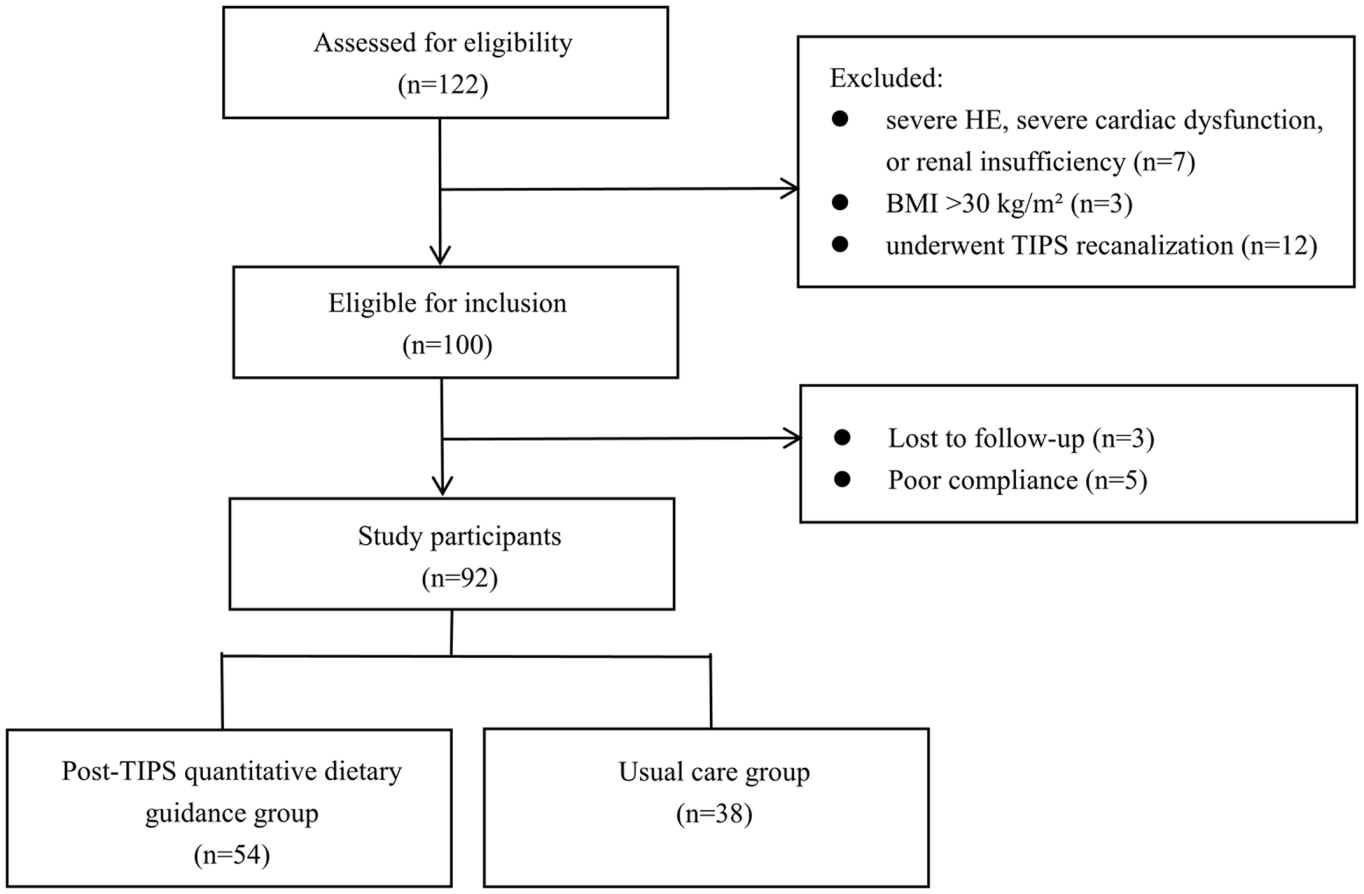
Flow chart. HE, hepatic encephalopathy; TIPS, transjugular intrahepatic portosystemic shunt.

Between April 2022 and September 2023, a total of 122 patients with liver cirrhosis met the eligibility criteria, of whom 92 were included in the final study (Figure 1). The baseline characteristics of the patients were shown in Table 1. Of the total cohort, 54 patients received post-TIPS individualized quantitative dietary guidance during their hospital stay, whereas 38 patients received usual care. In the usual care group, most reasons for declining the intervention were subjective and unrelated to baseline nutritional or clinical conditions. Compared with the usual care group, patients in the quantitative dietary guidance group showed no significant differences in age, gender, body height, body weight, dry body weight, adjusted BMI, etiology of liver cirrhosis, TIPS indications, NRS 2002, Child–Pugh classifications, Child–Pugh score, MELD score, skeletal muscle mass, SMI, sarcopenia, peripheral edema, degree of ascites, the puncture site of the portal vein, and stent size (all *p*>0.05). All baseline characteristics showed no significant difference between the two groups, supporting the validity of subsequent comparisons of prognostic indicators.

**Table 1:** Baseline characteristics.

Variable	Overall *N* =92	Usual care group *N* =38	Quantitative dietary guidance group *N* =54	*p*-value
Age	63.00 (54.00, 69.00)	64.00 (54.25, 68.00)	60.00 (53.00, 69.00)	0.36
Gender				0.96
Male (*n*, %)	53 (57.61%)	22 (57.89%)	31 (57.41%)	
Female (*n*, %)	39 (42.39%)	16 (42.11%)	23 (42.59%)	
Body height (m)	1.65±0.08	1.64±0.08	1.65±0.08	0.50
Body weight (kg)	57.25 (50.00, 66.00)	55.00 (50.00, 63.00)	60.00 (50.00, 68.00)	0.19
Dry body weight (kg)	53.27 (45.04, 61.64)	52.62 (45.01, 57.28)	54.00 (46.20, 64.75)	0.33
Adjusted BMI (kg/m^2^)	20.26±3.64	20.12±3.44	20.35±3.80	0.72
Etiology (*n*, %)				0.85
Virus	34 (36.96%)	14 (36.84%)	20 (37.04%)	
Alcoholic	11 (11.96%)	5 (13.16%)	6 (11.11%)	
Schistosoma	2 (2.17%)	0 (0.00%)	2 (3.70%)	
Others	45 (48.91%)	19 (50.00%)	26 (48.15%)	
TIPS indications (*n*, %)				0.42
Gastrointestinal bleeding	55 (59.78%)	22 (57.89%)	33 (61.11%)	
Refractory ascites	12 (13.04%)	7 (18.42%)	5 (9.26%)	
Others	25 (27.17%)	9 (23.68%)	16 (29.63%)	
Child–Pugh classifications (*n*, %)				0.94
A	31 (33.70%)	13 (34.21%)	18 (33.33%)	
B	50 (54.35%)	21 (55.26%)	29 (53.70%)	
C	11 (11.96%)	4 (10.53%)	7 (12.96%)	
Child–Pugh score	7.00 (6.00, 8.00)	7.00 (6.00, 8.00)	7.00 (6.00, 8.00)	0.73
MELD score	10.12 (8.08, 11.92)	10.08 (8.71, 11.01)	10.45 (8.05, 12.79)	0.63
Puncture site of portal vein (*n*, %)				0.67
Left branch	15 (16.30%)	7 (18.42%)	8 (14.81%)	
Right branch	10 (10.87%)	3 (7.89%)	7 (12.96%)	
Bifurcation	65 (70.65%)	28 (73.68%)	37 (68.52%)	
Unknown	2 (2.17%)	0 (0.00%)	2 (3.70%)	
Stent size (*n*, %)				0.88
6 (mm)	8 (8.70%)	4 (10.53%)	4 (7.41%)	
8 (mm)	84 (91.30%)	34 (89.47%)	50 (92.59%)	
NRS 2002 score	3.00 (3.00, 4.00)	3.00 (3.00, 4.00)	3.00 (3.00, 4.00)	0.67
Skeletal muscle mass (cm^2^)	99.08 (88.29, 105.59)	99.08 (84.88, 106.96)	99.08 (89.55, 104.59)	0.92
SMI (cm^2^/m^2^)	35.56±6.97	35.55±6.92	35.57±7.07	0.93
Sarcopenia (*n*, %)	68 (73.91%)	27 (71.05%)	41 (75.93%)	0.60
Peripheral edema (*n*, %)	11 (11.96%)	7 (18.42%)	4 (7.41%)	0.20
Ascites (*n*, %)				0.85
None	27 (29.35%)	13 (34.21%)	14 (25.93%)	
Mild	19 (20.65%)	7 (18.42%)	12 (22.22%)	
Moderate	30 (32.61%)	12 (31.58%)	18 (33.33%)	
Severe	16 (17.39%)	6 (15.79%)	10 (18.52%)	
Laboratory				
ALT (U/L)	22.00 (15.73, 36.95)	23.20 (12.07, 36.25)	22.00 (16.80, 37.42)	0.58
AST (U/L)	29.85 (21.05, 48.73)	29.85 (21.00, 47.92)	30.30 (21.18, 48.92)	0.88
Total bilirubin (μmol/L)	21.20 (12.75, 36.62)	16.40 (12.57, 31.70)	26.40 (14.35, 36.68)	0.12
Direct bilirubin (μmol/L)	7.30 (3.48, 14.38)	5.65 (3.42, 11.38)	9.00 (3.52, 16.95)	0.27
Albumin (g/L)	34.28±4.19	33.67±3.88	34.71±4.38	0.25
eGFR (mL/min/1.73m^2^)	114.00 (94.62, 135.78)	110.90 (78.57, 125.12)	116.40 (99.08, 148.50)	0.08
PT (seconds)	14.15 (13.28, 15.60)	14.15 (13.40, 15.47)	14.15 (13.15, 15.60)	0.71
INR	1.26 (1.18, 1.39)	1.27 (1.18, 1.38)	1.25 (1.17, 1.38)	0.80
Hemoglobin (g/L)	82.00 (72.00, 103.50)	85.50 (72.75, 106.50)	81.00 (72.00, 99.25)	0.47
CRP (mg/L)	6.50 (3.00, 21.05)	6.35 (2.85, 14.65)	7.55 (3.05, 22.48)	0.70
Serum ammonia (μmol/L)	51.05 (33.32, 73.00)	50.65 (35.60, 74.95)	51.55 (29.42, 71.22)	0.87

Data were presented as mean±standard deviation or median (quartile 1, quartile 3).

TIPS, transjugular intrahepatic portosystemic shunt; BMI, body mass index; NRS 2002, Nutritional Risk Screening; MELD, model for end-stage liver disease; SMI, skeletal muscle mass index; ALT, alanine aminotransferase; AST, aspartate aminotransferase; eGFR, estimated glomerular filtration rate; PT, prothrombin time; INR, international normalized ratio; CRP, C-reactive protein. ^*^p<0.05.

### 3.2 Post-TIPS adverse events during follow-up

As shown in Table 2, the incidence of OHE was significantly reduced in the quantitative dietary guidance group (12.96% vs. 36.84%, *p*=0.01). Liver-related death was also significantly lower in the quantitative dietary guidance group (1.85% vs. 15.79%, *p*=0.04), as was all-cause mortality (5.56% vs. 21.05%, *p*=0.05). No significant difference was observed in regard to the rates of uncontrolled rebleeding, liver transplantation, and stent dysfunction. The findings indicated that the group receiving quantitative dietary guidance demonstrated improved outcomes in terms of post-TIPS adverse events.

**Table 2:** Adverse events of cirrhotic patients who underwent TIPS.

Adverse events	Usual care group *N* =38	Quantitative dietary guidance group *N* =54	*p*-value
Uncontrolled rebleeding	4 (10.53%)	3 (5.56%)	0.63
OHE	14 (36.84%)	7 (12.96%)	0.01^*^
Liver transplantation	1 (2.63%)	1 (1.85%)	1.00
Liver-related death	6 (15.79%)	1 (1.85%)	0.04^*^
All causes of death	8 (21.05%)	3 (5.56%)	0.05^*^
Stent dysfunction (*n*, %)	1 (2.63%)	3 (5.56%)	0.87
Follow-up time (months)	24.57 (21.77, 30.17)	29.59 (23.63, 31.68)	0.18

TIPS, transjugular intrahepatic portosystemic shunt; OHE, overt hepatic encephalopathy. ^*^p<0.05.

### 3.3 Univariate and multivariate Cox regression analysis for liver-related mortality

Univariate analysis showed that quantitative dietary intervention was associated with reduced risk of mortality (HR 0.11, 95% CI 0.01–0.90, *p=* 0.04), while Child–Pugh score was associated with increased mortality risk (HR 1.58, 95% CI 1.04–2.39, *p=* 0.03). Age, gender, SMI, MELD score, TIPS indications, and concomitant tumor state showed no significant associations with mortality.

In multivariate analysis, quantitative dietary intervention was confirmed to be associated with decreased mortality risk (HR 0.09, 95% CI 0.01–0.75, *p*=0.03), while Child–Pugh score was associated with increased mortality risk (HR 1.70, 95% CI 1.10–2.64, *p=* 0.02). No other variables demonstrated significant associations in the adjusted model (Table 3).

**Table 3:** Cox regression analysis of factors associated with liver-related mortality.

	Univariate analysis			Multivariate analysis		
Variable	*B*	HR (95% CI)	*p*-value	*B*	HR (95% CI)	*p*-value
Age	0.00	1.00 (0.94, 1.07)	0.97			
Gender						
Male	Reference					
Female	0.64	1.89 (0.42, 8.44)	0.41			
Skeletal muscle mass index	−0.04	0.96 (0.86, 1.08)	0.49			
MELD score	0.10	1.11 (0.95, 1.30)	0.19			
Child–Pugh score	0.45	1.58 (1.04, 2.39)	0.03^*^	0.53	1.70 (1.10, 2.64)	0.02^*^
Dietary intervention						
No	Reference			Reference		
Yes	−2.23	0.11 (0.01, 0.90)	0.04^*^	−2.42	0.09 (0.01, 0.75)	0.03^*^
TIPS indications						
Gastrointestinal bleeding	Reference					
Refractory ascites	1.58	4.84 (0.68, 34.36)	0.12			
Others	1.27	3.54 (0.59, 21.20)	0.17			
Cancer	0.23	1.26 (0.15, 10.44)	0.83			

*B*, regression coefficient; HR, hazard ratio; CI, confidence interval; MELD, model for end-stage liver disease; TIPS, transjugular intrahepatic portosystemic shunt.

Multivariable Cox regression model. Candidate variables were selected from univariate analyses (*p*<0.1), including Child–Pugh score and dietary intervention. ^*^p<0.1.

### 3.4 Univariate and multivariate Cox regression analysis for OHE

Univariate analysis showed that age was associated with increased risk of OHE (HR 1.05, 95% CI 1.00–1.10, *p*=0.05). Quantitative dietary intervention (HR 0.32, 95% CI 0.13–0.80, *p*=0.01) and SMI (HR 0.94, 95% CI 0.88–1.01, *p*=0.08) were associated with decreased risk of OHE. Other factors showed no significant association with OHE.

In multivariate analysis, age was associated with increased risk of OHE (HR 1.06, 95% CI 1.00–1.12, *p*=0.04). Quantitative dietary intervention (HR 0.34, 95% CI 0.14–0.85, *p*=0.02) and SMI (HR 0.93, 95% CI 0.87–1.00, *p*=0.04) were associated with decreased risk of OHE (Table 4).

**Table 4:** Cox regression evaluation of factors associated with OHE.

	Univariate analysis			Multivariate analysis		
Variable	*B*	HR (95% CI)	*p*-value	*B*	HR (95% CI)	*p*-value
Age	0.05	1.05 (1.00, 1.10)	0.05^*^	0.06	1.06 (1.00, 1.12)	0.04^*^
Gender						
Male	Reference					
Female	−0.23	0.79 (0.33, 1.91)	0.60			
Skeletal muscle mass index	−0.06	0.94 (0.88, 1.01)	0.08^*^	−0.07	0.93 (0.87, 1.00)	0.04^*^
MELD score	0.01	1.01 (0.91, 1.13)	0.82			
Child–Pugh score	0.05	1.05 (0.82, 1.35)	0.72			
Dietary intervention						
No	Reference			Reference		
Yes	−1.14	0.32 (0.13, 0.80)	0.01^*^	−1.07	0.34 (0.14, 0.85)	0.02^*^
TIPS indications						
Gastrointestinal bleeding	Reference					
Refractory ascites	0.31	1.37 (0.45, 4.15)	0.58			
Others	−0.78	0.46 (0.13, 1.60)	0.22			
Cancer	−3.22	0.04 (0.00, 9.37)	0.25			

*B*, regression coefficient; HR, hazard ratio; CI, confidence interval; MELD, model for end-stage liver disease; TIPS, transjugular intrahepatic portosystemic shunt; OHE, overt hepatic encephalopathy.

Multivariable Cox regression model. Candidate variables were selected from univariate analyses (*p*<0.1), including skeletal muscle mass index, age, and dietary intervention. ^*^p<0.1.

### 3.5 Comparison of liver-related survival by Kaplan–Meier

**Figure 2:**
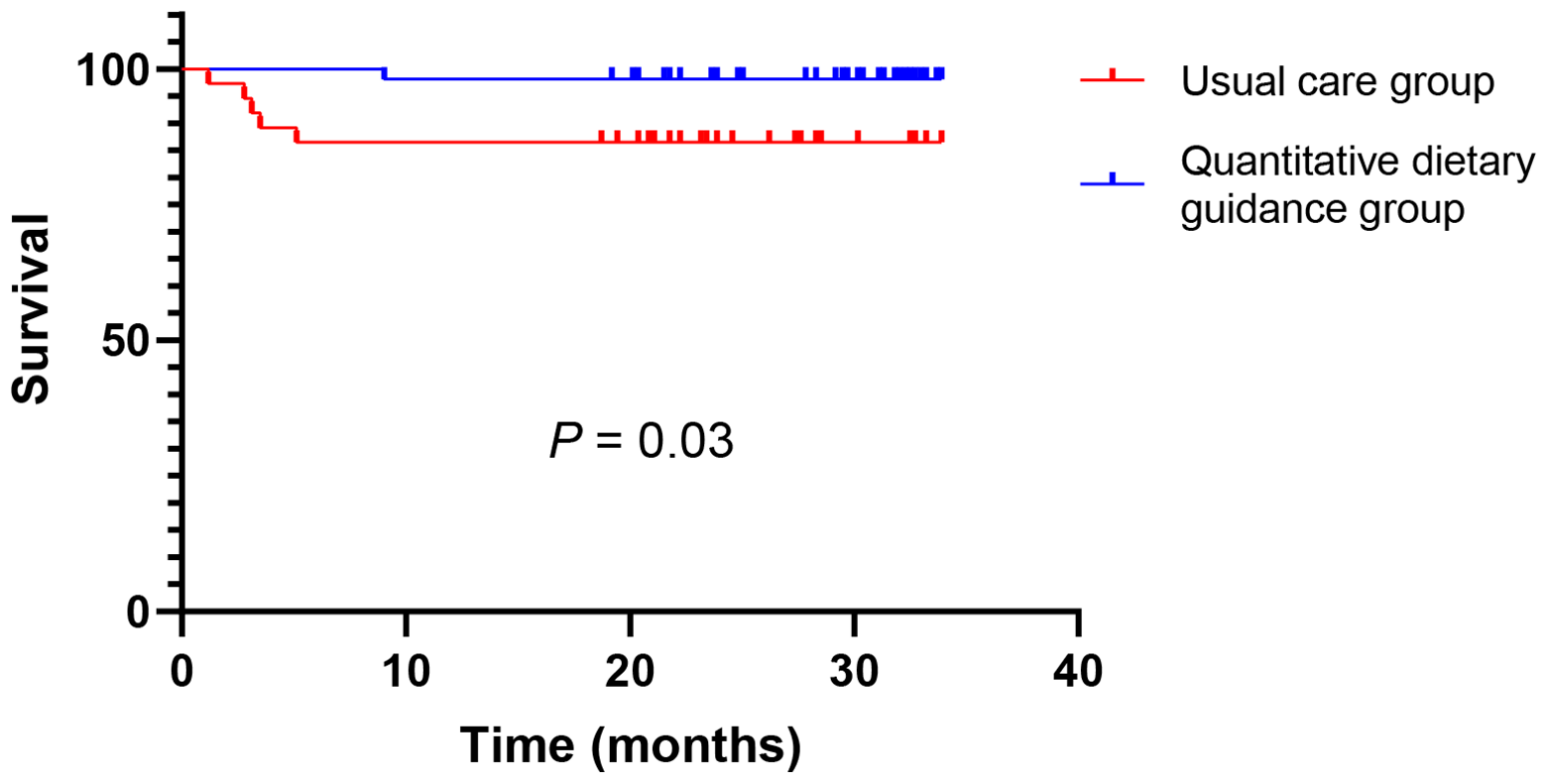
Liver-related survival analysis by Kaplan–Meier between two groups after TIPS during the follow-up.

As shown in Figure 2, survival was compared between groups after TIPS during follow-up. The liver-related mortality was significantly lower in the quantitative dietary guidance group compared to the usual care group (HR 0.13, 95% CI 0.02–0.66, *p*=0.03), indicating that patients in the quantitative dietary guidance group had a better prognosis in terms of liver-related survival.

### 3.6 Comparison of OHE by Kaplan–Meier

**Figure 3:**
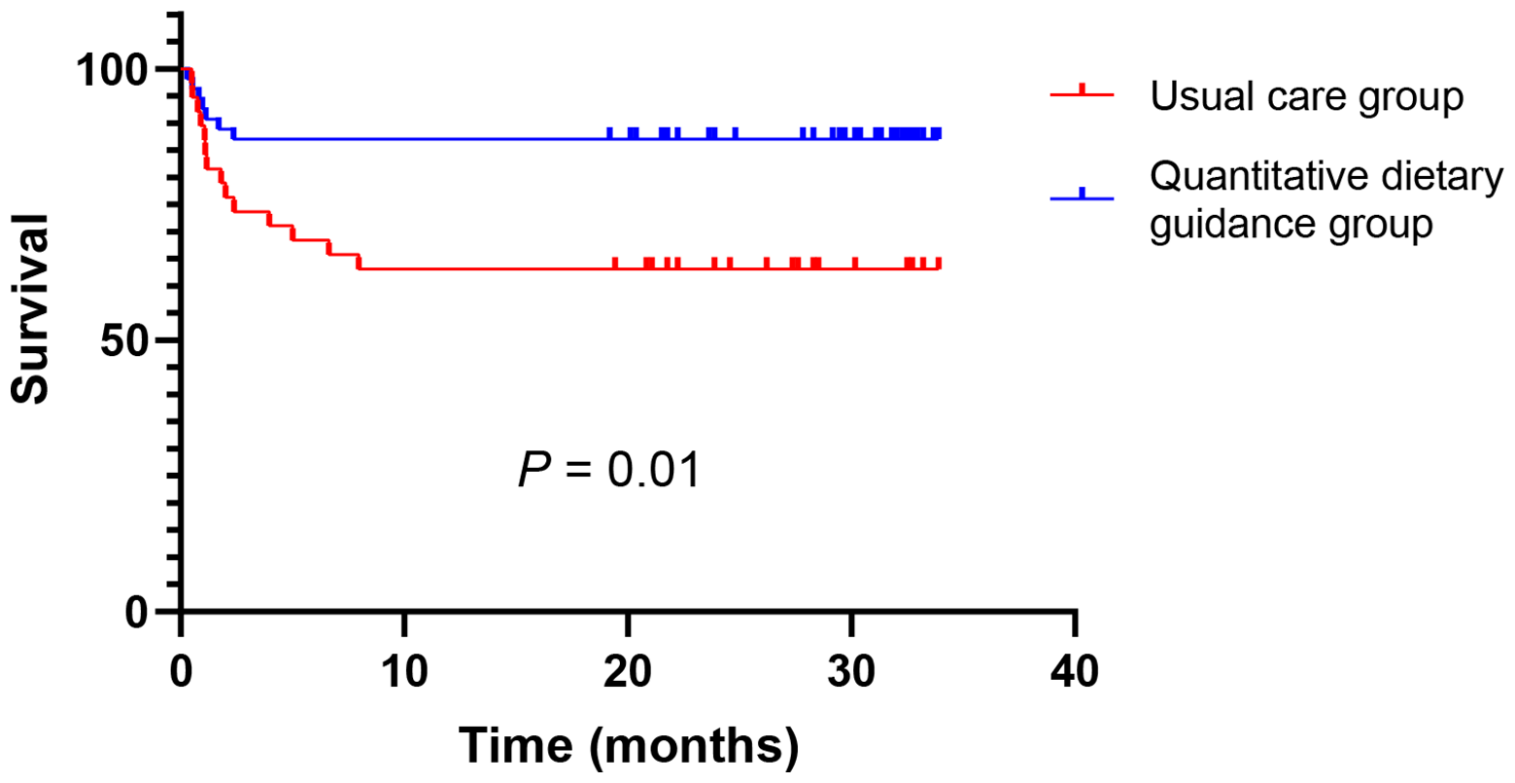
Comparison of OHE by Kaplan–Meier between two groups after TIPS during the follow-up.

As shown in Figure 3, the OHE probability was compared between groups after TIPS during follow-up. The OHE probability was significantly lower in the quantitative dietary guidance group compared to the usual care group (HR 0.32, 95% CI 0.13–0.77, *p*=0.01), indicating that patients in the quantitative dietary guidance group had a significantly lower risk of OHE during the follow-up compared to the usual care group.

## 4 Discussion

This study is the first to develop a quantitative dietary regimen specifically for patients after TIPS procedure and demonstrated that individualized, quantitative guidance for the perioperative period may significantly reduce both mortality and the incidence of OHE. This finding highlighted the importance of precise nutritional intervention in the early postoperative period of TIPS.

A hallmark metabolic disturbance in liver cirrhosis involved the dysregulation of energy substrate metabolism, characterized by accelerated proteolysis and progressive depletion of endogenous protein reserves. This pathophysiological alteration predisposed patients to protein-energy wasting, which might manifest even during the compensated stage (28). Some clinicians adapted protein-restricted diets to prevent postoperative OHE. Nevertheless, several studies suggested that low protein intake could lead to malnutrition and was associated with the incidence of HE and mortality in patients with cirrhosis (29–31). Conversely, excess protein intake might likewise increase the metabolic burden. An animal study noted that long-term intake of a high-protein diet resulted in a high acid load and increased liver triacylglycerol deposition pathways and hepatic signs of injury in rats (32). Therefore, balanced protein intake was key to improving prognosis. Adequate protein intake improved nutritional status in cirrhotic patients by maintaining homeostatic balance, facilitating hepatic functional recovery, and reducing the incidence of HE and mortality (33–36).

ESPEN guidelines recommended a protein intake of 1.2–1.5g/kg/day for patients with chronic liver disease (25). The TIPS procedure effectively reduced portal pressure by creating a portosystemic shunt, which allowed nitrogenous substances absorbed from the intestines to bypass hepatic metabolism and enter the systemic circulation directly, thereby significantly increasing the risk of hyperammonemia and OHE. In addition, liver function often experienced transient impairment during the early postoperative period, further compromising ammonia detoxification (37, 38). Notably, the short-term decline in liver function and the alterations in ammonia metabolism following TIPS left a gap in current guidelines for nutritional intervention during this critical period. The increased incidence of OHE and mortality at this stage remains a serious and challenging clinical issue. Based on previous clinical observations and the metabolic characteristics of post-TIPS patients, we developed a targeted dietary protocol limiting protein intake to 1.0g/kg/day during the first postoperative month, aiming to meet metabolic demands and avoid ammonia overload. The findings help give evidence to conduct further clinical trials in terms of this issue to validate the impact of the dietary regimen after TIPS. As one of the largest TIPS centers in the eastern region of China, our medical center had relatively sufficient TIPS cases, which helped us to better study this issue. This stage-specific regimen gradually transitioned to the guideline-recommended protein intake for chronic liver disease of 1.2–1.5g/kg/day, and contributed to the refined dietary management during the critical early period after TIPS.

Although nutritional guidelines for chronic liver disease had been established, the implementation had proven challenging for patients to follow due to a lack of specific, practical knowledge. Patients in the quantitative dietary guidance group were instructed comprehensively to quantify the weight of food on kitchen scales and combine it with individualized recipes, which significantly improved compliance. The beneficial effect of a tailored dietary instruction approach was demonstrated in several studies. Previous studies suggested that a quantitative high-protein nutrition intervention could help control insulin resistance and improve glucose metabolism in obese people (39, 40). Another study on non-alcoholic fatty liver disease showed that precision protein supplementation reduced liver fat and reduced hepatic necroinflammation (41). These studies suggested that translating guidelines into actionable quantitative programs was a core component of improving prognosis.

Similar with previous researches (42, 43), our study also found that SMI was a protective factor against OHE, indicating a significant association between muscle metabolism and HE. Skeletal muscles accounted for over 50% of ammonia removal (44). Protein restriction could exacerbate muscle loss, impaired ammonia metabolism, and elevate HE risk (45). Conversely, adequate protein intake could preserve muscle mass and reduce post-TIPS HE (42). Additionally, hyperammonemia perpetuated muscle wasting, thus creating a vicious cycle (46). Optimizing dietary protein might disrupt this cycle and prevent HE. In this study, the Child–Pugh score was associated with liver-related mortality, in accordance with previous studies (47, 48), confirming its critical role in prognostic evaluation for cirrhotic patients.

There were several limitations presented in the present study. First, this study was retrospective in design rather than randomized. Group allocation was determined by patients’ personal preferences rather than by random assignment, which may have introduced selection bias. Although most reasons for declining the intervention were subjective and unrelated to baseline characteristics, selection bias still cannot be completely excluded. Second, the relatively small sample size and single-center design might limit the generalizability of the findings. Although propensity score matching analysis could not be conducted due to limited sample size, the baseline characteristics between the two groups were generally balanced, still allowing for reasonable comparability. Furthermore, subgroup analyses were not performed due to the limited sample size. Future studies in larger cohorts are warranted to validate differential effects of nutritional intervention across subgroups. Additionally, the dietary intervention relied on self-reported compliance using kitchen scales, which may be subject to recall or reporting bias. Objective biomarkers or direct monitoring of dietary intake could strengthen future investigations. Due to the lack of robust preliminary data to inform effect size and feasibility, this study should be regarded as exploratory, providing initial real-world evidence. Future multicenter randomized controlled trials with larger cohorts, longer follow-up periods, and rigorous dietary monitoring are warranted to validate these findings and enhance their validity and translational impact.

## 5 Conclusion

We designed, for the first time, a nutritional guidance program specifically targeting post-TIPS patients. The study further demonstrated that providing individualized and quantitative dietary guidance during the perioperative period of TIPS could significantly reduce mortality and the incidence of OHE. Although the TIPS procedure could effectively reduce portal vein pressure, the increased risk of OHE remains clinically challenging. The present study may provide evidence to future prospective randomized studies to further validate the beneficial effect of the post-TIPS dietary regimen and provide basis for improving nutritional recommendations after TIPS procedure.

## Data Availability

The raw data supporting the conclusions of this article will be made available by corresponding authors upon reasonable request.

## References

[1] Rajesh S George T Philips CA Ahamed R Kumbar S Mohan N . Transjugular intrahepatic portosystemic shunt in cirrhosis: an exhaustive critical update. World J Gastroenterol. (2020) 26:5561–96. doi: 10.3748/wjg.v26.i37.5561, 33088154 PMC7545393

[2] Lee HL Lee SW. The role of transjugular intrahepatic portosystemic shunt in patients with portal hypertension: advantages and pitfalls. Clin Mol Hepatol. (2022) 28:121–34. doi: 10.3350/cmh.2021.0239, 34571587 PMC9013617

[3] Bozon-Riviere P Rudler M Weiss N Thabut D. Tips and hepatic encephalopathy in patients with cirrhosis. Metab Brain Dis. (2025) 40:117. doi: 10.1007/s11011-025-01541-w, 39903376

[4] Bureau C Thabut D Jezequel C Archambeaud I D’Alteroche L Dharancy S . The use of rifaximin in the prevention of overt hepatic encephalopathy after transjugular intrahepatic portosystemic shunt: a randomized controlled trial. Ann Intern Med. (2021) 174:633–40. doi: 10.7326/M20-0202, 33524293

[5] Larrue H Bureau C. Transjugular intrahepatic portosystemic shunt in portal hypertension: how to go further while staying on track? Hepatology. (2023) 77:344–6. doi: 10.1002/hep.32789 36106380

[6] Rose CF Amodio P Bajaj JS Dhiman RK Montagnese S Taylor-Robinson SD . Hepatic encephalopathy: novel insights into classification, pathophysiology and therapy. J Hepatol. (2020) 73:1526–47. doi: 10.1016/j.jhep.2020.07.013, 33097308

[7] Butterworth RF. Hepatic encephalopathy in cirrhosis: pathology and pathophysiology. Drugs. (2019) 79:17–21. doi: 10.1007/s40265-018-1017-0, 30706423 PMC6416236

[8] Ishikawa T Imai M Ko M Sato H Nozawa Y Sano T . Percutaneous transhepatic obliteration and percutaneous transhepatic sclerotherapy for intractable hepatic encephalopathy and gastric varices improves the hepatic function reserve. Biomed Rep. (2017) 6:99–102. doi: 10.3892/br.2016.811, 28123716 PMC5244787

[9] Simon-Talero M Roccarina D Martinez J Lampichler K Baiges A Low G . Association between portosystemic shunts and increased complications and mortality in patients with cirrhosis. Gastroenterology. (2018) 154:1694–705. doi: 10.1053/j.gastro.2018.01.028 29360462

[10] European Association for the Study of the Liver. EASL clinical practice guidelines on nutrition in chronic liver disease. J Hepatol. (2019) 70:172–93. doi: 10.1016/j.jhep.2018.06.024 30144956 PMC6657019

[11] Cabral CM Burns DL. Low-protein diets for hepatic encephalopathy debunked: let them eat steak. Nutr Clin Pract. (2011) 26:155–9. doi: 10.1177/0884533611400086, 21447768

[12] Bajaj JS Lauridsen M Tapper EB Duarte-Rojo A Rahimi RS Tandon P . Important unresolved questions in the management of hepatic encephalopathy: an ISHEN consensus. Am J Gastroenterol. (2020) 115:989–1002. doi: 10.14309/ajg.0000000000000603, 32618647

[13] Abou-Assi SG Mihas AA Gavis EA Gilles HS Haselbush A Levy JR . Safety of an immune-enhancing nutrition supplement in cirrhotic patients with history of encephalopathy. JPEN J Parenter Enteral Nutr. (2006) 30:91–6. doi: 10.1177/014860710603000291, 16517953

[14] Cordoba J Lopez-Hellin J Planas M Sabin P Sanpedro F Castro F . Normal protein diet for episodic hepatic encephalopathy: results of a randomized study. J Hepatol. (2004) 41:38–43. doi: 10.1016/j.jhep.2004.03.023, 15246205

[15] Tandon P Low G Mourtzakis M Zenith L Myers RP Abraldes JG . A model to identify sarcopenia in patients with cirrhosis. Clin Gastroenterol Hepatol. (2016) 14:1473–80. doi: 10.1016/j.cgh.2016.04.040 27189915

[16] Wu Y Zhu Y Feng Y Wang R Yao N Zhang M . Royal free hospital-nutritional prioritizing tool improves the prediction of malnutrition risk outcomes in liver cirrhosis patients compared with nutritional risk screening 2002. Br J Nutr. (2020) 124:1293–302. doi: 10.1017/S0007114520002366, 32600494 PMC7656665

[17] Xu X Ding H Li W Xu J Han Y Jia J . Chinese guidelines on the management of liver cirrhosis (abbreviated version). World J Gastroenterol. (2020) 26:7088–103. doi: 10.3748/wjg.v26.i45.7088, 33362370 PMC7723671

[18] Chen W Yuan Q Li X Yao J Yuan L Chen X . The role of sarcopenic obesity for the prediction of prognosis of patients with gastrointestinal cancer. Cancer Med. (2024) 13:e7452. doi: 10.1002/cam4.7452, 38953401 PMC11217812

[19] He Y Hu L Wu S Li L Zhong K Li J . Nutritional screening and assessment tools for patients with cirrhosis based on the global leadership initiative on malnutrition criteria. J Hum Nutr Diet. (2024) 37:430–9. doi: 10.1111/jhn.13265, 37932103

[20] He Y Wang Z Wu S Li L Li J Zhang Y . Screening and assessment of malnutrition in patients with liver cirrhosis. Front Nutr. (2024) 11:1398690. doi: 10.3389/fnut.2024.1398690, 39091687 PMC11292113

[21] Kikuchi N Uojima H Hidaka H Iwasaki S Wada N Kubota K . Prospective study for an independent predictor of prognosis in liver cirrhosis based on the new sarcopenia criteria produced by the Japan Society of Hepatology. Hepatol Res. (2021) 51:968–78. doi: 10.1111/hepr.13698, 34269502

[22] Nishikawa H Shiraki M Hiramatsu A Moriya K Hino K Nishiguchi S. Japan Society of Hepatology guidelines for sarcopenia in liver disease (1st edition): recommendation from the working group for creation of sarcopenia assessment criteria. Hepatol Res. (2016) 46:951–63. doi: 10.1111/hepr.12774 27481650

[23] Zhao L Tie J Wang G Li Z Xu J Zhuge Y . Efficacy of tips plus extrahepatic collateral embolisation in real-world data: a validation study. BMJ Open Gastroenterol. (2024) 11:e001310. doi: 10.1136/bmjgast-2023-001310, 38395452 PMC10895241

[24] Yin X Gu L Zhang M Yin Q Xiao J Wang Y . Covered tips procedure-related major complications: incidence, management and outcome from a single center. Front Med. (2022) 9:834106. doi: 10.3389/fmed.2022.834106, 35602500 PMC9116508

[25] Plauth M Bernal W Dasarathy S Merli M Plank LD Schutz T . ESPEN guideline on clinical nutrition in liver disease. Clin Nutr. (2019) 38:485–521. doi: 10.1016/j.clnu.2018.12.022, 30712783 PMC6686849

[26] Weissenborn K. Hepatic encephalopathy: definition, clinical grading and diagnostic principles. Drugs. (2019) 79:5–9. doi: 10.1007/s40265-018-1018-z, 30706420 PMC6416238

[27] Ridola L Faccioli J Nardelli S Gioia S Riggio O. Hepatic encephalopathy: diagnosis and management. J Transl Int Med. (2020) 8:210–9. doi: 10.2478/jtim-2020-0034, 33511048 PMC7805282

[28] Yao CK Fung J Chu NHS Tan VPY. Dietary interventions in liver cirrhosis. J Clin Gastroenterol. (2018) 52:663–73. doi: 10.1097/MCG.0000000000001071, 29912757

[29] Faron A Abu-Omar J Chang J Bohling N Sprinkart AM Attenberger U . Combination of fat-free muscle index and total spontaneous portosystemic shunt area identifies high-risk cirrhosis patients. Front Med. (2022) 9:831005. doi: 10.3389/fmed.2022.831005, 35492329 PMC9040492

[30] Praktiknjo M Simon-Talero M Romer J Roccarina D Martinez J Lampichler K . Total area of spontaneous portosystemic shunts independently predicts hepatic encephalopathy and mortality in liver cirrhosis. J Hepatol. (2020) 72:1140–50. doi: 10.1016/j.jhep.2019.12.021, 31954206

[31] Ney M Abraldes JG Ma M Belland D Harvey A Robbins S . Insufficient protein intake is associated with increased mortality in 630 patients with cirrhosis awaiting liver transplantation. Nutr Clin Pract. (2015) 30:530–6. doi: 10.1177/0884533614567716, 25667232

[32] Diaz-Rua R Keijer J Palou A van Schothorst EM Oliver P. Long-term intake of a high-protein diet increases liver triacylglycerol deposition pathways and hepatic signs of injury in rats. J Nutr Biochem. (2017) 46:39–48. doi: 10.1016/j.jnutbio.2017.04.008, 28454041

[33] Daftari G Tehrani AN Pashayee-Khamene F Karimi S Ahmadzadeh S Hekmatdoost A . Dietary protein intake and mortality among survivors of liver cirrhosis: a prospective cohort study. BMC Gastroenterol. (2023) 23:227. doi: 10.1186/s12876-023-02832-1, 37400778 PMC10316618

[34] Deng S Zhu Y Chen Z Li W. Application progress of early nutrition intervention in patients with hepatocellular carcinoma after liver transplantation. World J Gastrointest Surg. (2025) 17:100321. doi: 10.4240/wjgs.v17.i3.100321, 40162388 PMC11948105

[35] Ndraha S Simadibrata M. Normal protein diet and l-ornithine-l-aspartate for hepatic encephalopathy. Acta Med Indones. (2010) 42:158–61. 20724770

[36] Maharshi S Sharma BC Sachdeva S Srivastava S Sharma P. Efficacy of nutritional therapy for patients with cirrhosis and minimal hepatic encephalopathy in a randomized trial. Clin Gastroenterol Hepatol. (2016) 14:454–60. doi: 10.1016/j.cgh.2015.09.028, 26453952

[37] Li Y Wu Y Wu H. Management of hepatic encephalopathy following transjugular intrahepatic portosystemic shunts: current strategies and future directions. World J Gastroenterol. (2025) 31:103512. doi: 10.3748/wjg.v31.i15.103512, 40309228 PMC12038546

[38] Ahuja NK Ally WA Caldwell SH. Direct acting inhibitors of ammoniagenesis: a role in post-tips encephalopathy? Ann Hepatol. (2014) 13:179–86. 24552859

[39] Tettamanzi F Bagnardi V Louca P Nogal A Monti GS Mambrini SP . A high protein diet is more effective in improving insulin resistance and glycemic variability compared to a Mediterranean diet-a cross-over controlled inpatient dietary study. Nutrients. (2021) 13:4380. doi: 10.3390/nu13124380, 34959931 PMC8707429

[40] Rodrigo-Carbo C Madinaveitia-Nisarre L Perez-Calahorra S Gracia-Rubio I Cebollada A Galindo-Lalana C . Low-calorie, high-protein diets, regardless of protein source, improve glucose metabolism and cardiometabolic profiles in subjects with prediabetes or type 2 diabetes and overweight or obesity. Diabetes Obes Metab. (2025) 27:268–79. doi: 10.1111/dom.16013, 39420528 PMC11618321

[41] Markova M Pivovarova O Hornemann S Sucher S Frahnow T Wegner K . Isocaloric diets high in animal or plant protein reduce liver fat and inflammation in individuals with type 2 diabetes. Gastroenterology. (2017) 152:571–85. doi: 10.1053/j.gastro.2016.10.007 27765690

[42] Gioia S Merli M Nardelli S Lattanzi B Pitocchi F Ridola L . The modification of quantity and quality of muscle mass improves the cognitive impairment after tips. Liver Int. (2019) 39:871–7. doi: 10.1111/liv.14050, 30667572

[43] Zeng X Shi Z Yu J Wang L Luo Y Jin S . Sarcopenia as a prognostic predictor of liver cirrhosis: a multicentre study in China. J Cachexia Sarcopenia Muscle. (2021) 12:1948–58. doi: 10.1002/jcsm.12797, 34520115 PMC8718091

[44] Engelmann C Claria J Szabo G Bosch J Bernardi M. Pathophysiology of decompensated cirrhosis: portal hypertension, circulatory dysfunction, inflammation, metabolism and mitochondrial dysfunction. J Hepatol. (2021) 75:S49–66. doi: 10.1016/j.jhep.2021.01.002, 34039492 PMC9272511

[45] Tapper EB Parikh ND Sengupta N Mellinger J Ratz D Lok AS . A risk score to predict the development of hepatic encephalopathy in a population-based cohort of patients with cirrhosis. Hepatology. (2018) 68:1498–507. doi: 10.1002/hep.29628, 29091289

[46] Lattanzi B D’Ambrosio D Merli M. Hepatic encephalopathy and sarcopenia: two faces of the same metabolic alteration. J Clin Exp Hepatol. (2019) 9:125–30. doi: 10.1016/j.jceh.2018.04.007, 30765945 PMC6363954

[47] Conejo I Guardascione MA Tandon P Cachero A Castellote J Abraldes JG . Multicenter external validation of risk stratification criteria for patients with variceal bleeding. Clin Gastroenterol Hepatol. (2018) 16:132–9. doi: 10.1016/j.cgh.2017.04.042 28501536

[48] Lv Y Zuo L Zhu X Zhao J Xue H Jiang Z . Identifying optimal candidates for early tips among patients with cirrhosis and acute variceal bleeding: a multicentre observational study. Gut. (2019) 68:1297–310. doi: 10.1136/gutjnl-2018-317057, 30415233

