# Supplementary material for: Impact of quantitative dietary guidance on postoperative outcomes in patients undergoing transjugular intrahepatic portosystemic shunt surgery: a retrospective cohort study
Source: Front Nutr. 2026 Feb 11;13:1671392. doi: 10.3389/fnut.2026.1671392 (PMC12932184; doi:10.3389/fnut.2026.1671392)
Supplement: Supplementary file 1 [file Table_1.DOCX]

Supplementary Table 1 Example Meal Plans of the Recommended Nutritional Protocol for Patients after TIPS within the first month

| Quantitative Dietary Plan Based on Body Weight Categories | | | | |
| --- | --- | --- | --- | --- |
| Basic Information | Standard Body Weight | 50Kg | 60Kg | 70Kg |
| Energy | Total Energy (kcal/day) | 1750 | 2100 | 2450 |
|  | Dietary Energy (kcal/day) | 1375 | 1725 | 2075 |
|  | ONS Energy (kcal/day) | 375 | 375 | 375 |
|  | Energy Standard  (kcal/day) | 35 | 35 | 35 |
| Protein | Total Protein (g/day) | 50 | 60 | 70 |
|  | Dietary Protein (g/day) | 34 | 44 | 54 |
|  | ONS Protein (g/day) | 16 | 16 | 16 |
|  | Protein Standard  (g/kg/d) | 1.0 | 1.0 | 1.0 |
| Meal Plan | All listed weights represent raw edible portions unless otherwise specified (meat weights indicate cooked portions)  20g of vegetable oil throughout the day | | | |
| Breakfast | Wheat flour (Wheat bun) | 25g (35g) | 75g (105g) | 75g (105g) |
|  | Boiled egg | 30g | 60g | 60g |
|  | Low-fat milk | 120ml | 120ml | 250ml |
|  | Rape/Romaine lettuce | 75g | 75g | 75g |
| Morning Snack | Orange/Apple/Kiwifruit | 75g | 75g | 150g |
|  | TPF-DM | 167ml | 167ml | 167ml |
| Lunch | Rice (steamed) | 75g (225g) | 75g (225g) | 75g (225g) |
|  | Shrimp/Fish | – | – | 15g |
|  | Corn starch noodle/Potato starch noodle | 50g | 75g | 75g |
|  | Soybean curd | 50g | 50g | 50g |
|  | Winter melon/Zucchini | 125g | 125g | 125g |
|  | Rape/Romaine lettuce | 75g | 75g | 75g |
| Afternoon Snack | Orange/Apple/  Kiwifruit | 75g | 75g | 150g |
|  | TPF-DM | 167ml | 167ml | 167ml |
| Dinner | Corn starch noodle/Potato starch noodle | 50g | 75g | 125g |
|  | Chicken/Duck | – | – | – |
|  | Soybean curd slab | 15g | 15g | 15g |
|  | Chinese cabbage | 100g | 100g | 100g |
| Bedtime Snack | TPF-DM | 167ml | 167ml | 167ml |
|  | Bread | 35g | 35g | 35g |

Abbreviations: ONS, Oral Nutritional Supplement; TPF-DM, Enteral Nutritional Suspension**.**
